# Novel coronavirus seropositivity and related factors among healthcare workers at a university hospital during the prevaccination period: a cross-sectional study

**DOI:** 10.1186/s12941-021-00436-9

**Published:** 2021-04-27

**Authors:** Aziz Ogutlu, Oguz Karabay, Unal Erkorkmaz, Ertugrul Guclu, Seher Sen, Abdulkadir Aydin, Mehmet Koroglu

**Affiliations:** 1 Department of Infectious Diseases and Clinical Microbiology, Faculty of Medicine, Sakarya University, Sakarya, Turkey; 2 Department of Bioistatistics, Faculty of Medicine, Sakarya University, Sakarya, Turkey; 3 Infectious Diseases Unit, Nursing Services, Sakarya Training and Research Hospital, Sakarya, Turkey; 4 Department of Family Medicine, Faculty of Medicine, Sakarya University, Sakarya, Turkey; 5 Department of Clinical Microbiology, Faculty of Medicine, Sakarya University, Sakarya, Turkey

**Keywords:** COVID-19, Pandemic, Antibody

## Abstract

**Background:**

This study aimed to investigate the specific risk factors for the transmission of novel coronavirus (SARS-CoV-2) among healthcare workers in different campuses of a university hospital and to reveal the risk factors for antibody positivity.

**Methods:**

In this retrospective cross-sectional study, 2988 (82%) of 3620 healthcare workers in a university hospital participated. The coronavirus disease 2019 (COVID-19) antibody was investigated using serum from healthcare workers who underwent COVID-19 antibody testing. The antibody test results of the participants were evaluated based on their work campus, their profession and their workplace. The statistical significance level was p < 0.05 in all analyses.

**Results:**

Of the participants in this study, 108 (3.6%) were antibody positive, and 2880 (96.4%) were negative. Antibody positivity rates were greater in nurses compared with other healthcare workers (p < 0.001). Regarding workplace, antibody positivity was greater in those working in intensive care compared to those working in other locations (p < 0.001).

**Conclusions:**

Healthcare workers are at the highest risk of being infected with COVID-19. Those who have a higher risk of infection among healthcare workers and those working in high-risk areas should be vaccinated early and use personal protective equipment during the pandemic.

*Trial Registration*: Retrospective permission was obtained from both the local ethics committee and the Turkish Ministry of Health for this study (IRB No:71522473/050.01.04/370, Date: 05.20.2020).

## Background

Coronavirus disease 2019 (COVID-19) has affected many people worldwide. Infection by SARS-CoV-2 has caused disease in greater than 80 million people as of December 2020 and caused approximately two million deaths. The disease can easily spread from person to person in society by respiratory droplets (coughing, sneezing, speech, etc.) and close contact with an infected person. Additionally, the disease is transmitted by touching of the eyes, nose, or mouth with contaminated hands after contact with contaminated surfaces [1]. In healthcare facilities, airborne contamination occurs during aerosol-generating applications as well as droplet-induced or contact spread.

SARS-CoV-2 is highly contagious, and healthcare workers (HCWs) experience a significant risk of transmission when caring for suspected or certain COVID-19 patients [2]. Various reports show that many HCWs in many hospitals worldwide have been infected with SARS-CoV-2 [3]. Over time, the pandemic has seriously affected life in our country, and many HCWs fell ill or died during this epidemic. Health care workers have a higher risk of COVID-19 transmission than the community [4].

Many guidelines have been published to prevent HCWs from becoming infected [5]. Despite infection control measures, it is not sufficient to prevent the spread of SARS-CoV-2 among HCWs. Unknown risk factors can also contribute to virus transmission in hospitals. Despite all the precautions taken, HCWs continue to be infected. The most important way to determine the number of infected HCWs is to determine the frequency by serosurvey. This study aimed to examine the antibody distribution of those working in a university hospital in May 2020 and the relationship of antibody frequency according to profession and workplace.

## Methods

This retrospective cross-sectional study, was performed in a university hospital with 1200 inpatient beds, including 160 (adult: 100, neonatal:50, pediatric:10) intensive care unit beds. The hospital consists of four different campuses. After the first COVID-19 case was reported in Turkey (March 11), these four campuses, including the Central Campus and Toyota Campus, were transformed into pandemic hospitals. Maternity and Pediatrics Campus and Korucuk Campus continued their routine operation. This study was performed with healthcare workers in these hospitals in May 2020.

A total of 3620 HCWs were actively working on these four campuses as of May 2020. All HCWs working on four campuses were informed about the antibody screening to be performed at the hospital. A list of all HCWs was made, and each were given an appointment for blood sample collection. The appointments were determined by giving priority to the campuses and clinics where COVID-19 patients were treated. Antibody test results of 2988 HCWs whose blood samples were taken from among 3620 HCWs were evaluated. In this cross-sectional type of research, 82.5% of the participants were reached.

The first laboratory-confirmed COVID-19 case in Sakarya Province was detected on March 20. A flexible working model was created for all campuses. All HCWs were provided with personnel protective equipment that should be used while caring for COVID-19 patients. HCWs were trained on the use of personnel protective equipment. HCWs were asked to first put on an isolation gown, then a mask, then goggles or face shield, and finally gloves. These operations were requested to be performed in reverse order when taking off the equipment.

Blood collection teams were established in the hospital. In groups of two, these teams simultaneously obtained blood samples from participants working in intensive care and COVID-19 clinics to prevent contamination and minimize the density of HCWs in one area. A screening outpatient clinic was opened for those working in other units of campuses. Other blood samples were obtained at this outpatient clinic at the appointment times given to the HCWs. All blood samples taken were submitted to the sample acceptance unit.

The kit used [COVID-19 IgM/IgG Total Antibody Rapid Test (Beijing Hotgen Biotech Co. Ltd, China)] was based on the principle of colloidal gold immunochromatographic technology. The kit employs the double antigen sandwich method to detect SARS CoV-2 IgM/IgG total antibody levels in serum or plasma samples. Sensitivity and specificity rates have not been provided because these kits were produced in the early stages of the pandemic. However, regarding the performance characteristics of the kit, the positive reference coincidence rate, sensitivity reference coincidence rate, negative reference coincidence rate and repeatibility values were reported to be 100% [5].

Peripheral blood samples taken from the patient were studied from the sera after centrifugation at 4000 rpm/10 min. Studies were conducted in biosafety level 2 cabinets. After taking 10 µl of serum and adding the cassette to the well on the test, 3 drops of diluent were added. After 15 min of incubation at room temperature, the test result was evaluated within 30 min. Studies were conducted according to the manufacturer's recommendations.

### Statistical analysis

All categorical variables were compared using the chi-square test. The odds ratio and 95% confidence intervals for the odds ratio were calculated to determine the risk levels for their professions, workplace, and hospital of the HCWs in terms of SARS-CoV-2 positivity. Categorical variables are presented as frequencies and percentages. A p-value < 0.05 was considered significant. Analyses were performed using commercial software (IBM SPSS Statistics, Version 23.0. Armonk, NY: IBM Corp. and MedCalc Statistical Software version 19.6, MedCalc Software bvba, Ostend, Belgium).

Permission for this study was obtained from both the local ethics committee (IRB No: 71522473/050.01.04/370, Date: 05.20.2020) and the Turkish Ministry of Health.

## Results

In total, 2988 HCWs in our hospital were included in this study. A total of 72.8% of the employees were from the main campus, 16.3% were from maternity and pediatric campuses, and the remaining employees were from the Toyota 10.5% and Korucuk 0.4% campuses. The distribution of HCWs, their professions, and working places are summarized in Table 1. Of the personnel tested for antibodies, 108 (3.6%) were positive, whereas 2880 (96.4%) were antibody negative.

**Table 1:** Distribution of HCWs tested with suspicion of SARS-CoV-2 based on hospital, duty, and place of duty

Features		n	%
Hospitals	Center Hospital	2176	72.8
	Maternity and Pediatrics Hospital	486	16.3
	Toyota Hospital	315	10.5
	Korucuk Hospital	11	0.4
Profession	Medical Doctor	496	16.6
	Nurse	982	32.9
	Other Health Personnel	352	11.8
	Medical Secretary	311	10.4
	Cleaning staff	474	15.9
	Administrative/Technical Staff	265	8.9
	Security Guard	108	3.5
Workplace	Covıd-19 Clinics	202	6.8
	Intensive Care Units	595	19.9
	Emergency Services	381	12.8
	Clinics/Polyclinics	910	30.5
	Laboratories/Radiodiagnostic	264	8.8
	Administrative/Technical Units	445	14.8
	General	191	6.4

**Fig. 1:**
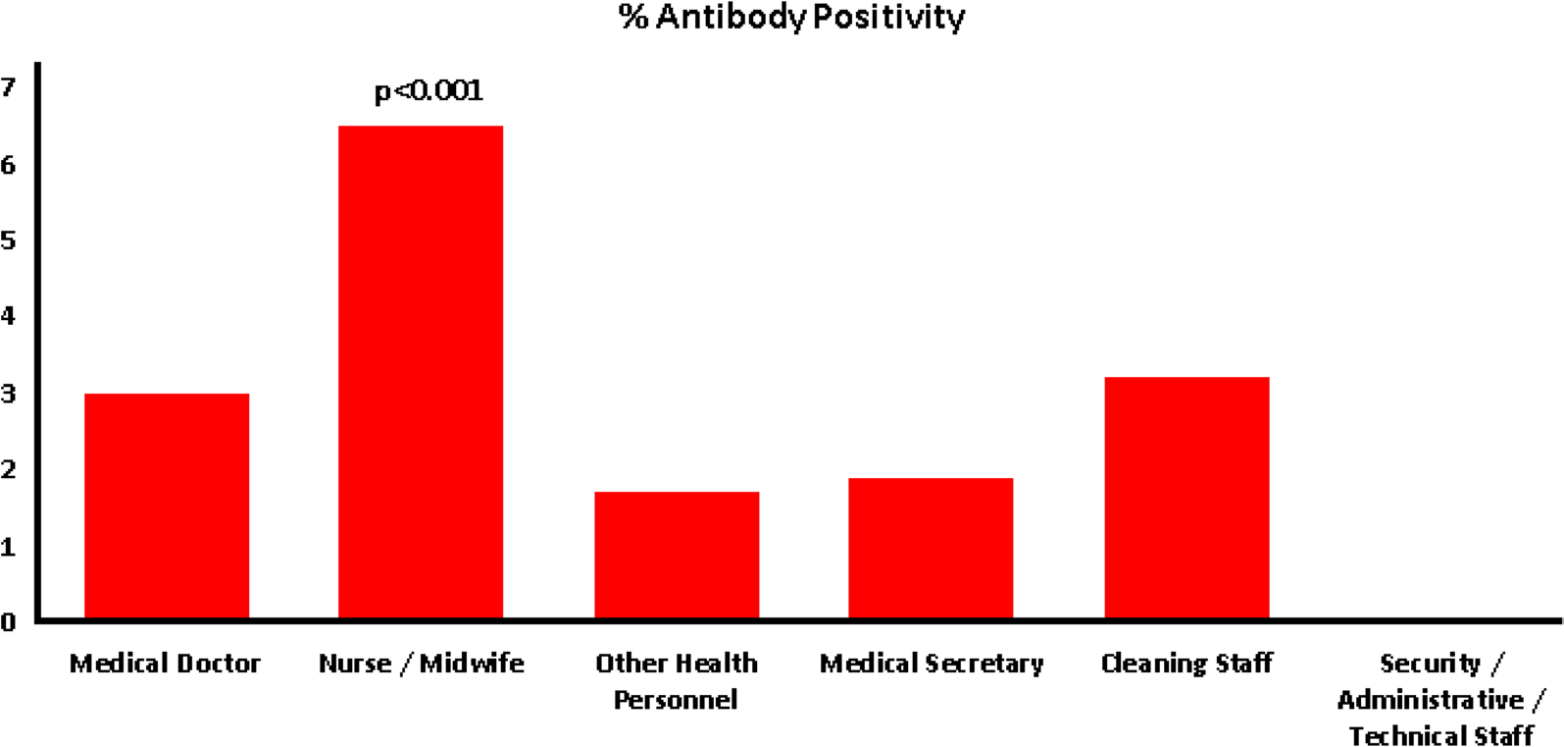
Antibody positivity based on the profession of HCWs

The probability of antibody positivity in nurses was 3.36 times higher than that in other occupational groups (Table 2). We found that among the staff groups, nurses were at the highest risk (Fig. 1; Table 3).

**Table 2:** Distribution of SARS-CoV-2 antibody positivity according to the professions, workplace, and hospitals in which healthcare professionals work

	Antibody Test Results		OR	95% CI for OR	P
	Negative (n = 2880)	Positive (n = 108)			
Profession					
Medical Doctor	481 (97.0%)	15 (3.0%)	0.804	0.462–1.,400	0.441
Nurse	916 (93.3%)	66 (6.7%)	3.369	2.270–5.000	< 0.001
Other Health Personnel	346 (98.3%)	6 (1.7%)	0.465	0.203–1.067	0.071
Medical Secretary	305 (98.1%)	6 (1.9%)	0.497	0.216–1.141	0.099
Cleaning Staff	459 (96.8%)	15 (3.2%)	0.851	0.489–1.481	0.567
Security/Administrative/Technical Staff	373 (100.0%)	0 (0.0%)	0.031	0.002–0.499	0.014
Work place					
COVID-19 Clinics	190 (94.1%)	12 (5.9%)	1.770	0.954–3.283	0.070
Intensive Care Units	540 (90.8%)	55 (9.2%)	4.497	3.049–6.633	< 0.001
Emergency Services	374 (98.2%)	7 (1.8%)	0.464	0.214–1.007	0.052
Clinics/Polyclinics	889 (97.7%)	21 (2.3%)	0.541	0.334–0.876	0.013
Laboratories/Radiodiagnostic	258 (97.7%)	6 (2.3%)	0.598	0.260–1.375	0.226
Administrative/Technical Units	439 (98.7%)	6 (1.3%)	0.327	0.143–0.750	0.009
General	190 (99.5%)	1 (0.5%)	0.132	0.018*–0.953	0.045
Hospitals					
Center Hospital	2081 (95.6%)	95 (4.4%)	2.806	1.563–5.038	< 0.001
Maternity and Pediatrics Hospital	482 (99.2%)	4 (0.8%)	0.191	0.070–0.522	0.001
Toyota Hospital	306 (97.1%)	9 (2.9%)	0.765	0.383–1.529	0.448
Korucuk Hospital	11 (100.0%)	0 (0.0%)	1.150	0.067–19.641	0.923
Hospitals					
Pandemic Hospitals	2387 (95.8%)	104 (4.2%)	5.370	1.969–14.646	0.001
Standard Hospitals	493 (99.2%)	4 (0.8%)			

*OR* odds ratio, *CI* confidence interval

^*^Odds ratios of each profession, workplace, and hospital were calculated according to all other professions, workplaces, and hospitals

**Table 3:** COVID-19 risk ratio of doctors, nurses and cleaning staff compared to other healthcare professionals

Professions	OR	95% CI for OR	p
Medical Doctor*			
Nurse	0.433	0.244–0.766	0.004
Other Health Personnel	1.798	0.691–4.682	0.229
Medical Secretary	1.585	0.608–4.130	0.346
Cleaning Staff	0.954	0.461–1.974	0.897
Security/Administrative/Technical Staff	24.047	1.434–403.207	0.027
Nurse*			
Medical Doctor	2.311	1.305–4.091	0.004
Other Health Personnel	4.155	1.785–9.671	0.001
Medical secretary	3.663	1.572–8.533	0.003
Cleaning staff	2.205	1.245–3.905	0.007
Security/Administrative/Technical Staff	54.201	3.346–877.929	0.005
Cleaning staff*			
Medical Doctor	1.048	0.507–2.168	0.897
Nurse	0.453	0.256–0.804	0.007
Other Health Personnel	1.885	0.724–4.907	0.194
Medical secretary	1.661	0.638–4.329	0.299
Security/Administrative/Technical Staff	25.198	1.503–422.541	0.025

*OR* odds ratio, *CI* confidence interval

^*^Odds ratios of medical doctors, nurses, and cleaning staffs were calculated according to related professions

## Discussion

In this study, the frequency of COVID-19 antibodies in hospital staff was investigated based on the tests performed as of May 2020. The results revealed a 3.6% antibody positivity rate in the staff. It was observed that nurses represent the riskiest group among HCWs. The probability of antibody positivity in nurses was 3.36-fold greater than that in other occupational groups. When antibody positivity is assessed based on the professions in the research, the detection of virus-specific antibodies indicates encounters with COVID-19. Antibodies generally reach detectable levels 1 to 2 weeks after infection. Therefore, antibody tests are not suitable to demonstrate acute infection [7]. In a study from the US, approximately 6% of adults hospitalized with COVID-19 were HCWs, and 72% of them were women. Greater than two-thirds of HCWs hospitalized with COVID-19 generally work in positions that involve direct contact with patients, and greater than one-third are nurses [8].

However, it should not be forgotten that a negative antibody test does not exclude infection. Antibody tests are mainly used in serological surveillance studies. Antibody tests are not suitable for use as immune indicators, and results do not suggest that preventive measures should be relaxed [6].

One study found that healthcare workers providing care to COVID-19 patients exhibited a greater risk of contracting COVID-19. The risk of hospitalization related to COVID-19 was compared among healthcare workers who provided health services to patients with COVID-19, other healthcare workers, household members of healthcare professionals and the general population. In the first 3 months, the probability of hospitalization with COVID-19 was increased three-fold in healthcare workers providing direct care for COVID-19 patients compared with other healthcare workers. In analyses adjusted for gender, age, ethnicity, socioeconomic status and comorbidity, the risk was twice as high among household members of employees who directly cared for a COVID-19 patient [9].

According to our findings, those working in intensive care units (9.2%) and those working in the COVID-19 service had the highest antibody positivity. Employees in this group care for more serious patients and are exposed to a more intense virus load. Therefore, this group of HCWs with high risk should be included in the first group of individuals to receive a COVID-19 vaccine. For individuals working in high-risk areas, such as emergency and intensive care, the use of personnel protective equipment should be maintained meticulously, and employees working in this field should not experience any shortages in equipment. Additionally, a limitation should be placed on the number of patients seen on a daily basis to reduce intense patient contact, and flexible work schedules should be created to reduce the virus load in HCWs. The rate of positivity in the households of infected HCWs, not only elderly HCWs, is also expected to be high. Considering this situation, households of HCWs should be included in the scope of screening.

In our country, COVID-19 positivity was examined in the general population months after the date of this research. In that study, the COVID-19 antibody positivity rate in the population was 0.81% in June 2020. However, the 3.6% rate we found in May 2020, when COVID-19 was limited in the population, was well above the average of the general population [10].

Nurses had the highest antibody positivity among healthcare workers. Most patients with COVID-19 who need hospitalization have significant dyspnea. For this reason, patients who need oxygen support and nursing care the most. Therefore, we think that the intensive work load of nurses and intense contact with these patients may be related to the increased antibody positivity in this occupational group. In a study conducted in Denmark, nurses had the highest antibody positivity rate [11]. Approximately one-tenth of the HCWs screened in this study were diagnosed with acute SARS-CoV-2 infection; approximately half of them were nurses. The high number of nurses who were positive for SARS-CoV-2 in our study can be explained by the fact that their nurses generally spend more time performing direct patient care. Nurses spend a longer amount of time working at the bedside, and nurses perform duties that require direct patient contact. In addition, SARS-CoV-2 infection may be more common in nurses because nurses have to eat during the working period and nurses get together more in social areas during break periods.

Burnout has also increased in HCWs due to the increased workload and difficult working hours. Increasing burnout causes negative effects on physical and mental health. Any measure to help reduce the burnout levels of HCWs can reduce stress levels and provide effective strategies to improve physical and mental health [12, 13].

COVID-19 positivity was assessed among healthcare workers in a study conducted in Switzerland, and 9.6% antibody positivity was found. However, the data we found in this study are approximately 3 times lower. The main reason for this situation is that the outbreak started later in our country compared with other countries, such as Switzerland and Italy. We believe that if we performed this study a few months later, we would observe higher antibody positivity rates [14].

According to the data obtained, 3% antibody positivity is noted in healthcare personnel before vaccination These data reveal how valuable and necessary it is to vaccinate all healthcare personnel. Today, healthcare personnel have been vaccinated in many countries (e.g., Israel, France and England). However, there are still many low-income countries that cannot vaccinate their risky populations and healthcare personnel. In this regard, humanity should engage in a joint effort, and a strategy should be devised to vaccinate all at-risk groups worldwide. In addition, antibodies generated in response to vaccination may not provide immunity against new variants, so humanity remains at risk. Therefore, regardless of vaccination status, all healthcare professionals and risk groups should continue to use personal protective equipment while working.

Before any conclusion is reached, we should state the limitations of our study. One of the limitations of our study was its retrospective design. In addition, we could not investigate age or sex. If we could compare these data with postvaccination data, we could have more effective interpretations.

## Conclusions

Antibody positivity is high in HCWs. Practices that encourage early isolation are required to prevent cross-infection. Among HCWs, nurses and intensive care workers exhibit the greatest risk of COVID-19 infection.

## Data Availability

The datasets generated and/or analyzed during the current study are not publicly available but are available from the corresponding author on reasonable request. The data are not available to the public because that antibody test results of healthcare workers can only be used with the permission of the Ministry of Health and the relevant hospital.

## Abbreviations

SARS-COV-2: Novel Coronavirus
COVID-19: Coronavirus Disease
HCWs: Healthcare Workers
